# Microarray and cDNA sequence analysis of transcription during nerve-dependent limb regeneration

**DOI:** 10.1186/1741-7007-7-1

**Published:** 2009-01-13

**Authors:** James R Monaghan, Leonard G Epp, Srikrishna Putta, Robert B Page, John A Walker, Chris K Beachy, Wei Zhu, Gerald M Pao, Inder M Verma, Tony Hunter, Susan V Bryant, David M Gardiner, Tim T Harkins, S Randal Voss

**Affiliations:** 1 Department of Biology and Spinal Cord and Brain Injury Research Center, University of Kentucky, Lexington, KY 40506, USA; 2 Department of Biology, Mount Union College, Alliance, OH 44601, USA; 3 Department of Biology, Minot State University, Minot, SD, USA; 4 The Salk Institute for Biological Studies, La Jolla, CA 92037, USA; 5 Department of Developmental and Cell Biology, University of California Irvine, Irvine, CA 92697, USA; 6 The Developmental Biology Center, University of California Irvine, Irvine, CA 92697, USA; 7 Roche Applied Science, Indianapolis, IN 46250, USA

## Abstract

**Background:**

Microarray analysis and 454 cDNA sequencing were used to investigate a centuries-old problem in regenerative biology: the basis of nerve-dependent limb regeneration in salamanders. Innervated (NR) and denervated (DL) forelimbs of Mexican axolotls were amputated and transcripts were sampled after 0, 5, and 14 days of regeneration.

**Results:**

Considerable similarity was observed between NR and DL transcriptional programs at 5 and 14 days post amputation (dpa). Genes with extracellular functions that are critical to wound healing were upregulated while muscle-specific genes were downregulated. Thus, many processes that are regulated during early limb regeneration do not depend upon nerve-derived factors. The majority of the transcriptional differences between NR and DL limbs were correlated with blastema formation; cell numbers increased in NR limbs after 5 dpa and this yielded distinct transcriptional signatures of cell proliferation in NR limbs at 14 dpa. These transcriptional signatures were not observed in DL limbs. Instead, gene expression changes within DL limbs suggest more diverse and protracted wound-healing responses. 454 cDNA sequencing complemented the microarray analysis by providing deeper sampling of transcriptional programs and associated biological processes. Assembly of new 454 cDNA sequences with existing expressed sequence tag (EST) contigs from the *Ambystoma *EST database more than doubled (3935 to 9411) the number of non-redundant human-*A. mexicanum *orthologous sequences.

**Conclusion:**

Many new candidate gene sequences were discovered for the first time and these will greatly enable future studies of wound healing, epigenetics, genome stability, and nerve-dependent blastema formation and outgrowth using the axolotl model.

## Background

Salamanders are fascinating vertebrate organisms because they routinely regenerate complex tissues. Within only a few weeks of losing a piece of limb to a hungry predator or scalpel-wielding scientist, a salamander perfectly reforms the missing structure. In the early history of salamander regeneration research, scientists innovated elegant experimental designs to probe the anatomical basis of regeneration [1]. More recently and in parallel with the discovery of conserved regulatory genes and developmental pathways among metazoans, scientists have focused attention on candidate molecules and signaling pathways whose functions were deduced first from studies of model organisms. In particular, much research has been devoted to understanding aspects of limb regeneration associated with wound healing that recapitulate limb development; this strategy has yielded many useful insights and molecular probes [2-14]. Although it is clear that key regulatory molecules play important roles in the development of all organisms, it is not clear that a framework for understanding regeneration can be constructed using a generic and limited molecular toolkit. There is a need to go beyond candidate molecules and use unbiased approaches to characterize the molecular complexity underlying salamander regeneration.

Recent research resource development for the Mexican axolotl now allows classic regeneration experiments to be re-examined with powerful and unbiased genomic approaches. One particularly elegant experiment performed almost 200 years ago showed that salamander limb regeneration requires the presence of peripheral nerves. Todd [15] found in 1823 that limb regeneration does not occur if the sciatic nerve of the hindlimb is severed shortly before or immediately after a more distal limb amputation. Subsequent research showed that the brachial nerves entering the forelimb are required to promote limb outgrowth and patterning of a new limb [16,17]. Within a few days of limb amputation, cells proximal to the amputation plane dedifferentiate and accumulate to form a blastema. Blastema cells proliferate and progressively differentiate during regeneration to give rise to all mesodermal structures of a typical vertebrate limb [18]. Upon amputation, nerve fibers in the vicinity of the amputation plane extend into the blastema and play a supportive role in cell proliferation [11,19]. Transection of the spinal nerves that enter the limb in the axolotl leads to a decrease in cycling cells and there is resorption of distal tissues of the amputated limb. Histological and cell proliferation analyses suggest that early cellular events are similar between denervated and innervated limbs, but denervated limbs are incapable of blastema formation because they do not support significant cell proliferation and outgrowth [20-25].

Although limb regeneration is a complex developmental process, nerve dependency and other aspects of regeneration have often been conceptualized as having a simple molecular basis, involving relatively few regulatory factors. For example, Ferretti and Brockes [26] hypothesized that in the absence of nerves, Schwann cells produce an inhibitory factor that prevents blastema cell proliferation. This mechanism is supported by experimental results although the hypothetical factor has not been identified [27,28]. Alternative mechanisms for nerve dependency have also been proposed for several factors with growth promoting effects [29-33]. A recent study identified newt anterior gradient protein (nAG) as a blastema cell growth-promoting factor *in vitro*, whose over-expression was sufficient to rescue regeneration of denervated and amputated limbs *in vivo *[34]. Most recently, nerve-dependent expression of the transcription factor *sp9 *has been identified as an early marker of dedifferentiation of the wound epithelium and the initiation of limb regeneration [11]. While considerable progress has been made in investigating the functions of candidate regulatory factors and signaling pathways, a broader systems-level perspective is needed to understand why multiple aspects of limb regeneration are dependent upon the presence of a nerve.

Genomic tools are now available that allow global characterization of the regeneration process in salamanders. Expressed sequence tag (EST) information has facilitated the development of an *Ambystoma *salamander Affymetrix microarray platform [35-38]. This platform and a high-throughput 454 cDNA sequencing approach were used in this study to compare transcript abundance among uninjured limbs, regenerating limbs, and limbs denervated at the time of amputation. The results show that innervated (NR) and denervated (DL) limbs exhibited similar (but not identical) gene expression patterns at five days post amputation (dpa) but then diverged as a blastema formed under the influence of nerves. The results are discussed within the context of previous studies of nerve dependency, highlighting specific genes and biological processes that are associated with blastema formation and outgrowth, and more generally, the Mexican axolotl's unparalleled ability to regenerate limbs.

## Results

### Morphology and histology of denervated and innervated limbs

**Figure 1:**
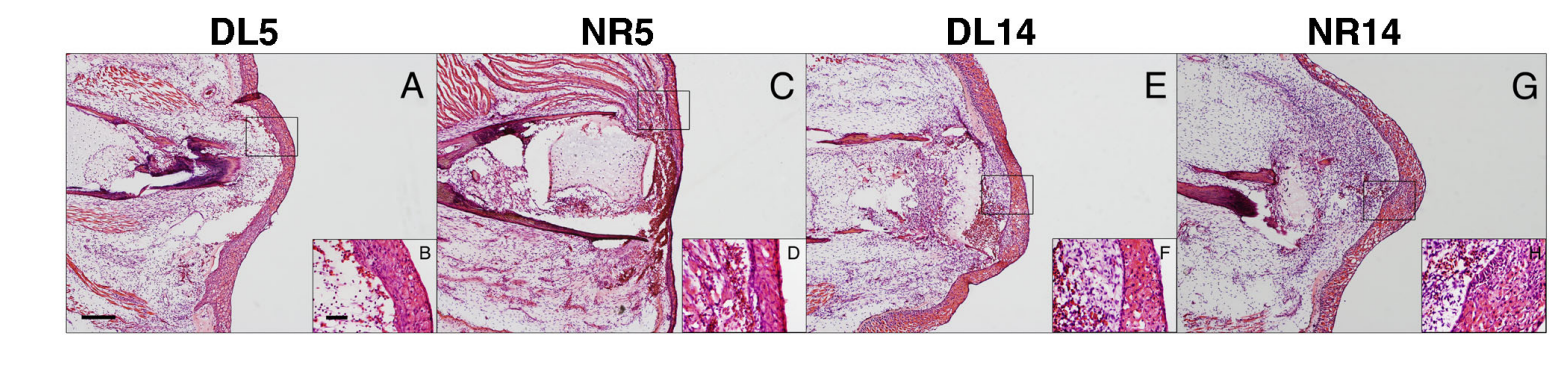
**Histology of innervated and denervated limbs at 5 and 14 dpa**. Eosin and hematoxylin staining of DL5 (A), NR5 (C), DL14 (E), and NR14 (G) limbs. Higher magnification inset pictures are provided for each image (B, D, F, H). Scale Bar A = 500 μm; B = 50 μm.

Histological staining verified our experimental procedures for creating innervated and denervated limbs on the same individual. Previous studies found few morphological or histological differences between innervated and denervated limbs during the first few days of regeneration [16,39,40]. Consistent with these observations, denervated and innervated limbs at 5 dpa (DL5 and NR5) were histologically indistinguishable (Figure 1a and 1c). Histological staining (hematoxylin and eosin) showed hemorrhaging directly beneath the epithelium containing Leydig cells, squamous cells, and basal keratinocytes (Figure 1b and 1d). All 5 dpa limbs resembled the wound-healing phase or early phase of dedifferentiation according to the staging of [41]. A blastema was visible on innervated limbs 14 dpa (NR14) and histological staining showed an accumulation of blastemal cells under the wound epithelium. These structures were not observed in denervated limbs at 14 dpa (DL14; Figure 1e to 1h). These results indicate that blastema formation and outgrowth only occurred in NR limbs. An apical thickening of the epithelium characterized the distal end of NR14 limbs and this layer consisted of keratinocytes and Leydig cells. These histological traits indicate that 14 dpa limbs in this experiment corresponded to the early to mid bud stage of limb regeneration [41]. Immunological staining using RT-97 was performed to detect the presence or absence of nerve axons at 5 and 14 dpa. At both time-points, neurofilament staining was positive in the NR limbs, but negative in DL limbs. In pilot experiments, we determined that > 20 days is required for nerves to re-innervate limbs after denervation surgery (data not shown). Thus, DL and NR limbs were created successfully and histology showed that 5 and 14 dpa time points correspond to wound-healing and early to mid bud phases of regeneration, respectively.

### Transcription during normal limb regeneration: Deviations of NR limbs from baseline

**Figure 2:**
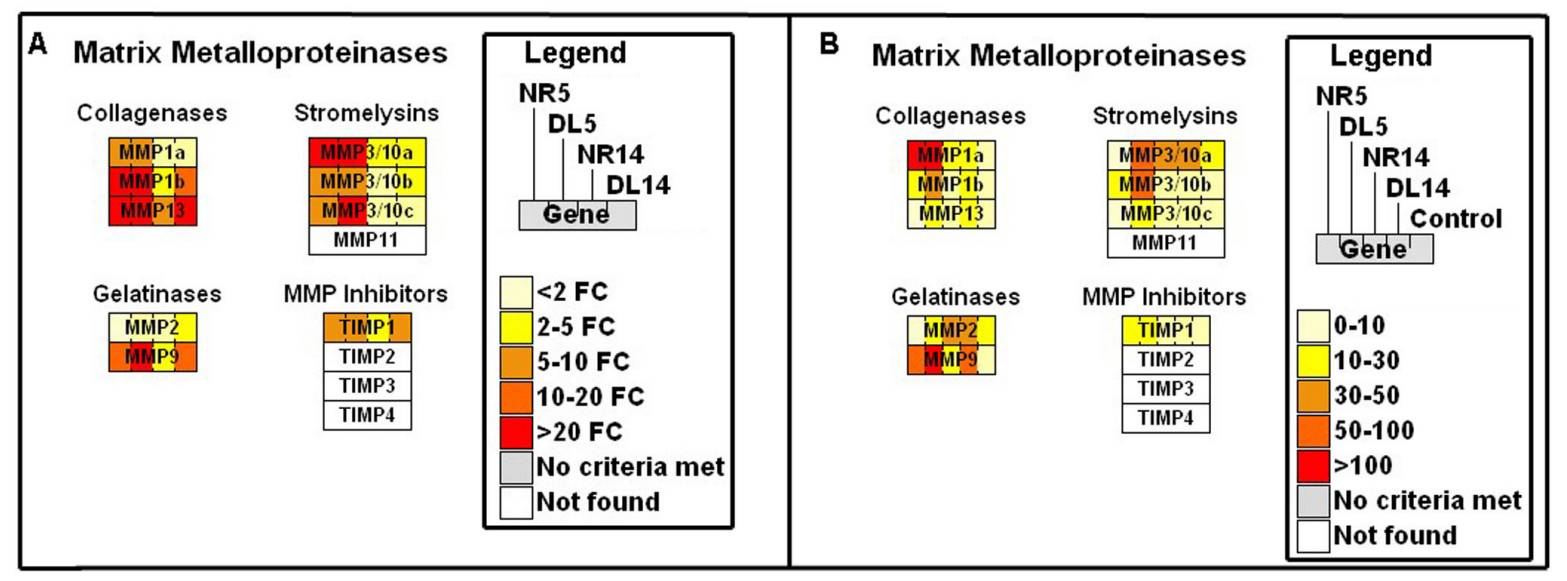
**Schematic of matrix metalloproteinase gene expression**. Matrix metalloproteinase gene expression is represented in each box. A) Microarray results are represented by fold change (FC) from day 0. B) Normalized counts are represented from the 454 cDNA sequencing experiment. Figures 2 to 4 were created using GenMAPP [101]. Figure 2 was modified from a MAPP originally created by Gladstone Institutes.

**Figure 3:**
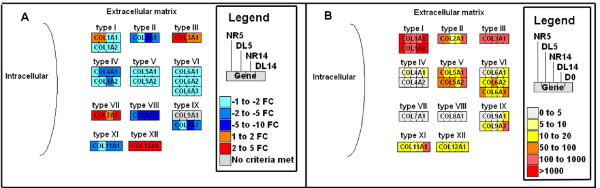
**Schematic of collagen gene expression**. Collagen gene expression is represented in each box. A) Microarray results are represented by fold change (FC) from day 0. B) Normalized counts are represented from the 454 cDNA sequencing experiment.

**Figure 4:**
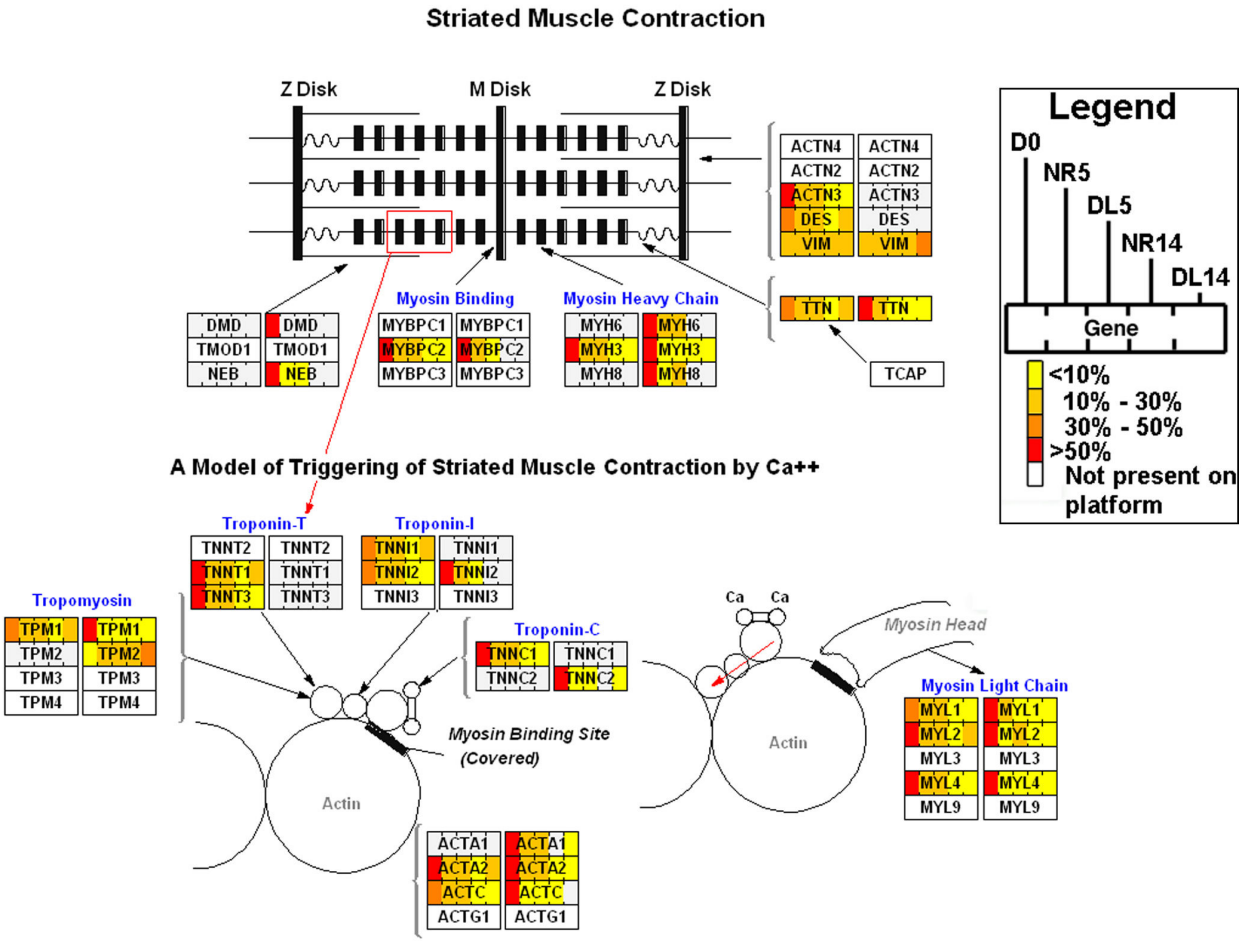
**Schematic of down-regulated muscle contraction genes**. Striated muscle contraction genes that were downregulated during limb regeneration. Each gene is represented by two boxes that denote proportional expression among Day 0, NR5, DL5, NR14, and DL14 samples. The left box reports hybridization intensity from the microarray experiment and the right box reports normalized count data from the 454 cDNA sequencing experiment. Figure 4 was modified from a MAPP originally created by Joanna Fong and Nathan Salomonis.

At both 5 and 14 dpa, mRNA levels for hundreds of genes were significantly different from baseline levels of genes expressed in whole limbs at Day 0 (Table 1). Many of the same genes (*n *= 215; up = 111; down = 104) were identified as significant in NR5 and NR14 limbs; the deviation from baseline was in the same direction for all but one of these genes (*fabp2*). Four matrix metalloproteinases (*mmp1*, *mmp3/10a*, *mmp*9, and *mmp13*) that are known to function during wound healing in many organisms were upregulated at both 5 and 14 dpa (Figure 2; Additional file 1), while collagens (*col4a1, col4a2*, *col8a1*, *col9a3*, and *col11a1*; Figure 3) and muscle-specific genes (Figure 4) were downregulated (Table 1; Additional file 2). Upregulation of collagen catabolism genes coupled with downregulation of collagen structural genes suggests that transcriptional activation and repression are integrated to efficiently remodel the extracellular environment of damaged tissues. Downregulation of muscle genes at both time points suggests that the differentiated muscle gene expression phenotype changes by 5 dpa, and changes more dramatically by 14 dpa.

**Table 1:** Significant gene ontology terms for changed genes

**upregulated**	**downregulated**
	

**NR5 and NR14 (N = 111)**	**NR5 and NR14 (N = 104)**

extracellular region (N = 14; 9.05E-07)	muscle contraction (N = 13; 1.81E-10)
collagen catabolism (N = 5; 4.35E-06)	cytoplasm (N = 51; 8.41E-07)
	collagen (N = 6; 4.05E-05)
	ion transport (N = 12; 9.44E-06)

**NR5 only (N = 110)**	**NR5 only (N = 49)**

extracellular region (N = 15; 1.23E-05)	extrinsic to membrane (N = 3; 6.10E-03)
response to stimulus (N = 17; 1.00E-02)	
signal transduction (N = 21; 2.89E-03)	
Ion transport (N = 9; 2.57E-03)	

**NR14 only (N = 53)**	**NR14 only (N = 93)**

DNA metabolic process (N = 8; 4.41E-04)	muscle contraction (N = 18; 5.43E-19)
	calcium ion binding (N = 11; 4.98E-04)

**DL5 and DL14 (N = 170)**	**DL5 and DL14 (N = 109)**

extracellular region (N = 23; 6.64E-10)	muscle contraction (N = 15; 6.62E-13)
lysosome (N = 11; 3.04E-08)	cytoplasm (N = 53; 1.46E-06)
ion transport (N = 13; 1.42E-05)	mitochondrial membrane (N = 12; 3.49E-04)
hydrolase activity (N = 26; 3.60E-05)	
response to stress (N = 21; 1.02E-03)	
signal transduction (N = 22; 5.40E-03)	
collagen catabolism (N = 5; 5.09E-05)	

**DL5 only (N = 68)**	**DL5 only (N = 58)**

extracellular region (N = 9; 3.68E-04)	extrinsic to membrane (N = 3; 4.64E-03)
response to stimulus (N = 12; 3.70E-03)	

**DL14 only (N = 69)**	**DL14 only (N = 111)**

extracellular region (N = 7; 4.10E-03)	muscle contraction (N = 9; 4.26E-05)
immune response (N = 5; 7.80E-03)	mitochondrion (N = 27; 6.71E-07)
	cytoplasmic (N = 43; 2.58E-03)
	M phase (N = 9; 1.40E-03)
	glucose metabolic process (N = 6; 2.97E-03)

Significant genes were identified by microarray analysis. Gene ontology terms are represented that were sampled more often than expected in NR5, NR14, DL5, and DL14 limbs. *P*-value is calculated by DAVID [45].

Although many genes exhibited similar deviations from baseline at 5 and 14 dpa, unique gene expression changes were identified at each time point (Table 1; Additional files 1 and 2). The unique NR5 genes were associated with gene ontology (GO) terms that implicate extracellular protein changes and signal transduction pathways of the early wounding response. These terms included response to stimulus, signal transduction, extracellular region, and ion transport. The unique NR14 genes were associated with GO terms that implicate cell division and DNA metabolism including *ccnd2*, *ccnb1*, *rrm1*, *rrm2*, *nasp*, *rrc1*, and *cdc20*. The unique gene expression changes that were identified at 5 and 14 dpa support the idea of temporal progression from an early wound-healing phase to a blastema outgrowth phase during normal limb regeneration.

### Transcription within denervated limbs: Deviations of DL limbs from baseline

As was observed in NR limbs, hundreds of genes were identified as significant when comparing DL5 and DL14 mRNA levels with baseline levels measured at Day 0 (Table 1; Additional files 1 and 2). Some of the same or similar GO terms that were associated with NR limbs were identified as significantly enriched in DL limbs. This was not unexpected because both DL and NR limbs undergo tissue histolysis at the limb stump and carry out an early wound-healing response (Figure 1). For example, extracellular region and MMP genes were upregulated at 5 and 14 dpa, as was seen in NR limbs (Figure 2; Additional file 1). As in NR limbs, muscle contraction (Figure 4), cytoplasmic, and collagen genes (Figure 3; *col4a1*, *col4a2*, *col8a1*) were downregulated in both DL5 and DL14 limbs (Additional file 2). Moreover, 83% of the genes that were identified as significant in NR5 limbs were also significant (and in the same direction) in DL5 limbs. These results indicate that many transcriptional events are nerve-independent during early regeneration.

Denervated limbs are known to cease growth following limb amputation. Several groups of genes may explain this observation including the downregulation of genes associated with the M phase of cell division, mitochondrial transcripts, and genes associated with glucose metabolism (Table 1; Additional file 2). Furthermore, DL14 limbs were morphologically similar to DL5 limbs because denervation prevented blastema formation (Figure 1). Consistent with this observation, many of the genes that were identified as upregulated in DL5 limbs were also identified as significant in DL14 limbs (69%). These genes are associated with the following GO terms: lysosome, response to stress, hydrolyse activity, signal transducer activity, and ion transport. Overall, these terms suggest protraction and expansion of wound-healing responses in DL14 limbs, as well as changes in cellular metabolism and cell division.

### Transcript abundance differences between NR and DL limbs

In the preceding two sections, transcriptional patterns of NR and DL limbs were described relative to baseline levels at Day 0. Here, we report genes found to be significant when comparing NR and DL limbs. In general, few significant changes in gene expression were observed when NR5 and DL5 transcripts were compared directly. Transcripts for 16 genes were more abundant in NR5 limbs and 17 were more abundant in DL5 limbs, and these differences were small in terms of fold-level change (< 3-fold difference between NR5 and DL5; Additional file 3). Eighteen of these genes exhibited significant sequence similarity to human or salamander presumptive gene sequences; the others are unknown. The genes with significantly more transcripts in NR5 limbs are associated with intracellular (for example *dnase1l3, uap1*, *acy3*, *nans*, *myl4*) and extracellular functions, including membrane proteins (for example *psca, umod*, and *emp1*) and collagen binding (*serpinh1*). The genes with significantly more transcripts in DL5 limbs are associated with extracellular functions or the immune response (for example *igll1*, *CD74*, *hmox1*, *neil1*, *marco*, *sftpd*, *mmp9*, *mrc1*). All but one of the significant 5 dpa genes showed the same directional deviation at 14 dpa; *myl1*was 1.5-fold higher in the NR limb at 5 dpa, but 2.2-fold lower at 14 dpa. Thus, as was observed when comparing DL5 and NR5 mRNA abundances with baseline levels, relatively few gene expression differences were identified between NR and DL limbs at 5 dpa, and the magnitude of these differences was small. These results further support the idea that transcription during limb regeneration is predominantly nerve-independent at 5 dpa.

**Figure 5:**
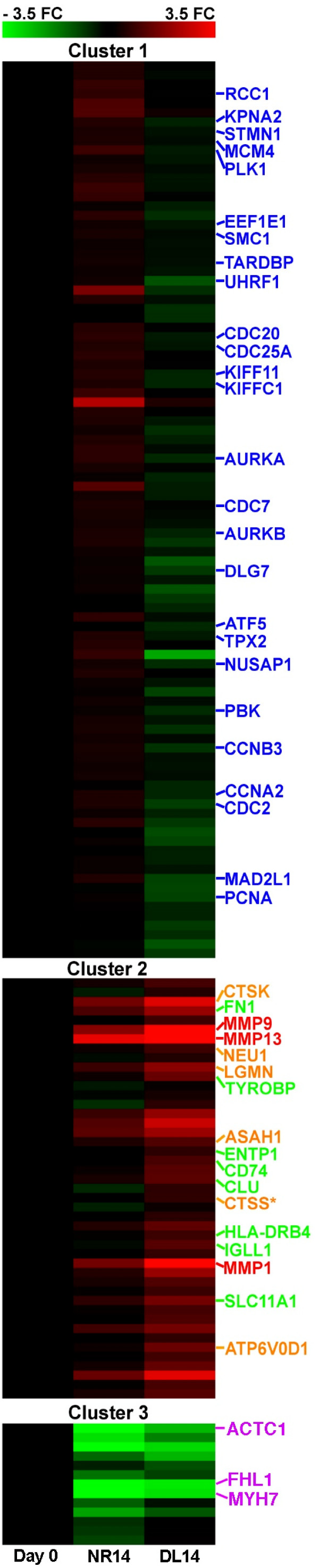
**Clustering of genes identified as significant from the comparison of NR14 and DL14 limbs**. Fold change values are relative to baseline levels at Day 0. Blue-coded genes are cell cycle associated; orange-coded genes localize to the lysosome; green-coded genes are associated with inflammatory responses; red-coded genes are matrix metalloproteinases; purple-coded genes are associated with muscle. Genes coded * are associated with inflammation and localize to the lysosome.

Whereas only 33 transcriptional differences were observed between morphologically similar DL and NR limbs at 5 dpa, 282 differences were detected at 14 dpa (Additional file 4). *K*-means cluster analysis of these genes with significant human protein hits highlighted three clusters wherein genes exhibited similar patterns of expression (Figure 5). Genes in Cluster 1 presented expression patterns with transcript abundances above baseline in NR14 limbs and abundances below baseline levels in DL14 limbs. Genes associated with cell cycling, a well-established characteristic of blastemal cells, were highly enriched in Cluster 1 (*n *= 26). Over 50% of the genes in Cluster 1 are predicted to localize to the nucleus, including several transcriptional regulators (*msx2*, *id3*, *tmpo*, *atf5*, *rbm15*, *spen*, *parp1*, and *tardbp*). Thus, many of the genes in Cluster 1 have functions that are consistent with blastema formation and outgrowth in NR limbs.

Genes from Clusters 2 and 3 were generally expressed in the same direction between NR14 and DL14 limbs; however, the magnitude of expression differed. Genes in Cluster 2 presented transcript abundances that generally exceeded baseline levels, with higher levels observed in DL14 limbs. This pattern suggests that most of these genes were activated in the same direction in the presence or absence of nerves, but denervation caused higher transcript abundances. Twenty-two percent of the genes in Cluster 2 are associated with the GO term cellular response to stimulus (*n *= 14) and a significant proportion localized to the lysosome (*n *= 6), including *lgmn*, *ctsk*, *ctss*, *asah*, *atp6v0d1*, and *neu1*. Genes in Cluster 3 presented transcript abundances that were generally lower than baseline levels, with much lower levels observed in NR14 limbs. With relatively few genes in this cluster, no biological process was identified as significantly enriched. However, inspection of the genes in Cluster 3 again supports the idea that some genes may function in muscle contraction (*actc1*, *myh7*, and *fhl1*) and tissue repair (*hsp27 *and *hebp2*). In summary, mRNA levels for genes from Clusters 2 and 3 were quantitatively affected by the presence or absence of a nerve.

### 454 cDNA sequence analysis of nerve dependency

**Figure 6:**
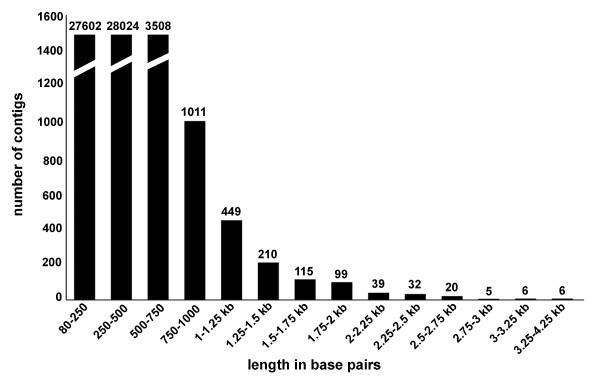
**Bar graph showing contig lengths**. Distribution of sequence lengths for each of the 61,127 contigs.

**Figure 7:**
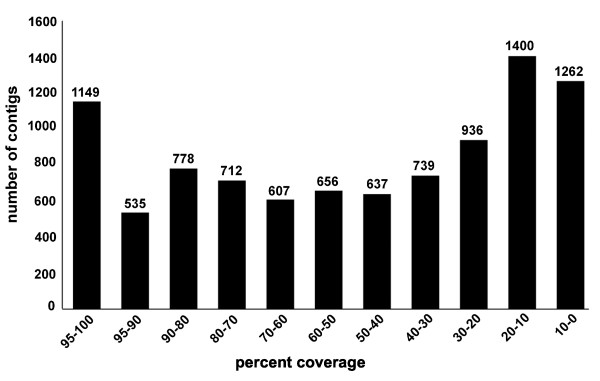
**Bar graph showing percent coverage of human proteins**. The distribution of the percent coverage for each of the unique 9411 human proteins with presumptive salamander orthologs.

To further explore nerve dependency and generate an unbiased collection of molecular probes for regenerating limbs, we sequenced cDNAs derived from the same RNA samples that were used in the microarray analysis. Over 1.7 × 10^6 ^reads were generated and this yielded approximately 90,000 to 230,000 high-quality sequence reads for each limb treatment, with an average of 215 base pairs in length (Table 2). More than half of the sequence reads correspond to mitochondrial transcripts and ribosomal RNA. This frequency of mtDNA transcripts (30%) approximates the number sampled in an earlier EST screen of the *Ambystoma *genome [42]. The number of rRNA transcripts was higher than expected. Assembly of all high-quality cDNA reads yielded 429,086 unique sequences. These sequences were assembled with previous EST contigs to produce 61,127 contigs each containing at least two overlapping sequences. The distribution of contig lengths is shown in Figure 6. All contigs and singletons were searched against NCBI databases to identify significant similarity matches that would suggest presumptive gene identities. *Ambystoma *contigs and singletons yielded 25,446 significant hits to sequences in the human RefSeq database (BLASTx, *e *< 1 × 10^-7^), including 9411 unique human genes. Figure 7 shows the distribution of percent coverage to predicted human RefSeq proteins. Interestingly, 7130 *Ambystoma *queries that did not show significant amino acid sequence identity to a human reference sequence did show significant nucleotide identity to a *Xenopus *sequence. Assembly of new 454 cDNA sequences with existing EST contigs from the *Ambystoma *EST database more than doubled (3935 to 9411) the number of non-redundant human-*A. mexicanum *orthologous sequences. This increase in sequence content was even among 10 GO functional categories that are relevant to salamander wound healing and regeneration (Table 3). Assuming that many of the anonymous 454 contigs and singletons (> 300,000) that were generated correspond to functional genes, significantly more than 10,000 different transcripts are expressed during the first two weeks of axolotl limb regeneration.

**Table 2:** 454 DNA sequence reads that were generated for each cDNA limb library

	**Day 0**	**NR5**	**DL5**	**NR14**	**DL14**	**totals**
**Total Reads**	312258	216281	220561	393012	578787	1720899
**Filtered**	19459	14390	18918	22679	88387	163833
**mtRNA/rRNA**	168954	111490	92136	171046	256684	800310
**Final Reads**	123845	90401	109507	199287	233716	756756

**Table 3:** Gene ontology breakdown of the 9411 salamander genes with presumptive human orthologs

**Ontology**	**Level**	**Old annotations**	**New annotations**
**cell cycle**	BP3	226	438
**metabolism**	BP2	1889	3982
**cell motility**	BP3	62	126
**mophogenesis**	BP2	109	272
**development**	BP1	342	795
**response to stress**	BP2	219	469
**cell death**	BP3	142	312
**catalytic activity**	MF1	1275	2855
**transcription factor activity**	MF2	112	344
**kinase activity**	MF4	159	456

		**Total (3935)**	**Total (9411)**

The table shows the number of *Ambystoma *ESTdb contigs with gene ontology annotations before and after 454 DNA sequencing. The total is less than the sum of each category because several genes may belong to multiple categories. BP = biological process. MF = molecular function.

### mRNA abundance estimates and gene discovery from 454 cDNA sequence data

The number of times that a non-redundant transcript was sampled by 454 cDNA sequencing was used to estimate mRNA abundances. The transcript counts for 1150 *Ambystoma *EST contigs (genes) differed significantly among limb cDNA pools that were created for each of the limb types (Additional file 5). It was possible to assign a putative ortholog to 563 of these genes; the remaining genes were considered anonymous. This final list of genes was compared with the significant gene lists from the microarray analysis. It was determined that for 271 of the 1150 significant genes from the 454 cDNA sequencing analysis, a portion of the gene sequence was represented by a probe set on the *Ambystoma *GeneChip. Of these, 104 genes were identified as significant by both methodologies and mRNA abundances for these genes were highly positively correlated (Additional file 6; median Spearman's correlation = 0.87). The 167 genes found to be significant by 454 cDNA sequencing, but not by microarray analysis, were mostly characterized by low fold changes from baseline (median fold change as estimated from 454 cDNA sequencing counts = 1.75); or conversely, registered low hybridization intensities in the microarray analysis (median rank among 4844 probe sets = 343).

Gene functions that were identified as significantly enriched by microarray analysis were also identified as significant by 454 cDNA sequencing. For example, the muscle contraction GO term was identified as highly enriched and the underlying genes were similarly downregulated relative to baseline (Table 4; Figure 4; Additional file 6). Also, genes sampled most often from NR14 limbs were associated with DNA metabolism, a biological process associated with cell cycling (Table 4), and transcripts for genes associated with cell proliferation and cell cycle progression (for example *pcna, smc1, ctps*, *umod*, *psca*, *smc1l1*, *rad21*) were either sampled more often among NR limbs or were only sampled from NR limbs (Additional file 5). Thus, 454 cDNA sequencing also identified genes in NR limbs that are consistent with blastema formation. Several functional terms that were not identified by microarray analysis were identified as enriched by 454 cDNA sequencing. Transcripts for 40 genes associated with macromolecule metabolism were most abundant in NR5 limbs compared with other limbs. Transcripts for 12 genes associated with macromolecule catabolism were most abundant in DL5 limbs, including *mmp1*, *mmp3/10a*, *mmp3/10b*, *mmp9*, and *mmp13*. This suggests that the presence or absence of nerves differentially affects transcriptional responses and regulation at 5 dpa. Also, 454 cDNA sequencing identified additional *psca*-like genes that were not represented on the GeneChip, and these were also differentially expressed between NR5 and DL5 limbs. These and other examples suggest that 454 cDNA sequencing complemented the microarray analysis by providing deeper sampling of transcriptional programs and associated biological processes. This revealed more candidate nerve-dependent gene expression changes at the earlier 5 dpa time point than was revealed by microarray analysis.

**Table 4:** Gene ontology results from 454 sequencing

**cDNA library**	**Most significant GO term**	**p-value**
Day 0 (N = 127)	striated muscle contraction (N = 17)	1.75E-27
NR5 (N = 84)	macromolecule metabolism (N = 40)	1.37E-05
DL5 (N = 59)	macromolecule catabolism (N = 12)	6.21E-08
NR14 (N = 54)	DNA metabolism (N = 6)	2.00E-02
DL14 (N = 43)	protein biosynthesis (N = 14)	1.44E-17

Gene ontology analysis of 465 genes with presumptive human orthologs identified by 454 DNA sequencing. Only the most significant gene ontology category was selected for the table.

In addition to providing estimates of mRNA abundance, 454 cDNA sequencing also discovered new gene sequences for the Mexican axolotl. These include genes that are known to affect developmental processes in other vertebrate models: *notch (1,2,3,4), nrg1*, *bmp1, wnt4, ctnna1, btnna1, dkk1, axin 1, nrg1, fgf10*, *sirt *(*1,2,5,6*), *stats *(*1, 2, 3, 5, 6*), *sema4f*, *tf*, *sfrp (1,2,5), rara, rarg*, *rxr, pdgf*, *acvrI *(*IB*, *IIA*, *IIB*), *bmprI*, *bmprII*, *smad (1*, *2*, *4*, *5*, *7*, *9*), *efna1*, *ntn1*, *slit2, slit3*, *robo1*, and *robo2*. Most of these genes were sampled less than 10 times and thus appear to be expressed at low levels. Other developmental genes that have not been previously associated with limb regeneration were sampled many more times than these candidates, and for the following examples, counts varied significantly among limb cDNA libraries. These include *mdk*, *fliI*, *tagln2*, *ddx5*, *umod*, and *cnot1 *(Additional file 5). Also, numerous retroelement-associated sequences were sampled differentially between DL and NR limbs (Table 5). Overall, the 454 sequencing approach verified the primary results from the microarray analysis and identified many new candidate genes and functional pathways that are associated with limb regeneration.

**Table 5:** Significantly changed retrovirus-like genes

**Contig**	**Best hit**	**Gene Name**	**NR5**	**DL5**	**NR14**	**DL14**	**C**
10177_Contig1	XP_001201693	rev. transcriptase-like	10	2	0	0	0
10435_Contig1	ABB52637	envelope polyprotein	19	7	0	2	0
10953_Contig8	NP_055405	end. retroviral w	5	15	0	1	0
109875_Contig1	AAD19348	rev. transcriptase-like	0	0	10	0	0
110157_Contig5	NP_079357	hyp. prot. LOC57523	7	20	42	6	0
126954_Contig17	AAM34208	polyprotein	12	22	32	6	8
12813_Contig1	NP_003409	zinc finger protein 9	14	37	67	20	37
154450_Contig16	XP_945963	retrotransposon-like 1	5	27	11	2	10
174946_Contig1	BAC82626	pol-like protein	0	0	11	0	0
185474_Contig140	XP_790598	Helitron transposon	81	276	314	106	59
195627_Contig1	XP_692835	gypsy 1 polyprot.	0	0	11	0	0
218328_Contig1	XP_945963	retrotransposon-like 1	0	0	13	0	0
2194_Contig1	XP_001345076	pol polyprotein	17	0	0	0	0
23276_Contig1	NP_006798	chromobox hom. 1	14	0	0	0	0
23893_Contig1	ZP_01758189	NBB2750 hyp. protein	12	0	0	0	0
32735_Contig1	XP_001201693	rev. transcriptase-like	12	0	0	0	0
331680_Contig1	XP_001336840	lambda-recomb.-like	2	12	30	11	6
352970_Contig1	AAD02928	rev. transcriptase	0	0	0	0	12
39722_Contig1	XP_687309	rev. transcriptase	98	117	138	51	92
42238_Contig456	NP_055883	paternally exp. 10	41	2	10	3	12
42238_Contig60	NP_055883	paternally exp. 10	7	35	37	10	27
89909_Contig1	BAD72127	rev. transcriptase	0	12	0	0	0
94735_Contig1	XP_001336840	lambda-recomb.-like	0	12	0	0	0

Genes identified as differentially expressed by 454 sequencing analysis that have high sequence similarity to retrovirus genes.

## Discussion

Microarray analysis and 454 cDNA sequencing were used to identify nerve-dependent and independent gene expression changes during limb regeneration in the Mexican axolotl. The results show that limb regeneration is associated with thousands of transcriptional changes. Considerable similarity was observed between the DL and NR transcriptional programs at 5 and 14 dpa. For example, genes that are critical to wound healing were upregulated in both limb types (Table 6) while genes that are associated with muscle structure and function were downregulated (Figure 4). Many of the transcriptional changes that were observed at 5 dpa were also observed at 14 dpa. Thus, many aspects of early limb regeneration are accomplished in the absence of nerves. However, gene expression differences were identified between DL and NR limbs at 5 and 14 dpa. Many of the transcriptional differences correlated with blastema formation; cell numbers increased in NR limbs after 5 dpa and this yielded a distinct transcriptional signature of cell proliferation in NR14 limbs. Overall, this study identified genes that are associated with wound healing, early events of blastema formation, and subsequent blastema cell proliferation and outgrowth. Below, we discuss and expand upon these primary results and highlight genes whose functions appear to be important for understanding the basis of nerve dependency and limb regeneration.

**Table 6:** Significantly changed wound-healing genes

**Probe ID**	**Best Hit**	**Symbol**	**NR5/C**	**DL5/C**	**NR14/C**	**DL14/C**	**Function**
SRV_01351_at	NP_002220	JUNB	4.60	4.74	2.35	3.46	stress resp.
SRV_10702_at	NP_000388	CYBB	2.84	4.11	1.73	2.46	stress resp.
SRV_00330_at	NP_000388	CYBB	2.39	3.46	1.58	2.23	stress resp.
SRV_03054_at	NP_006761	MARCO	2.00	3.11	1.83	3.01	stress resp.
SRV_00130_a_at	NP_000032	APOE	1.90	2.49	2.36	4.28	stress resp.
SRV_00442_at	NP_000569	SLC11A1	1.95	2.76	1.58	3.00	stress resp.
SRV_01821_at	NP_937758	TYROBP	1.98	2.45		2.65	stress resp.
SRV_03023_a_at	NP_006696	GADD45G	1.96	2.29		2.01	stress resp.
SRV_02588_a_at	NP_005558	LGALS3BP	1.91	2.37		1.59	stress resp.
SRV_01177_a_at	NP_001091645	ENTPD1	2.39	2.23		1.58	stress resp.
SRV_02586_at	NP_005558	LGALS3BP	1.79	2.25		1.81	stress resp.
SRV_11767_a_at	NP_001020466	IFITM5	1.75	1.63	1.56		stress resp.
SRV_02724_at	NP_005902	MAT2A	2.60	1.60	1.78		stress resp.
SRV_01617_a_at	NP_002843	PTX3	4.30	3.78			stress resp.
SRV_02002_at	NP_001020366	CES1	2.36	3.01			stress resp.
SRV_03221_a_at	NP_060049	DMBT1	1.81	2.50			stress resp.
SRV_07036_at	NP_000567	IL1B	1.70	1.53			stress resp.
SRV_02516_at	NP_005338	HSPA5	1.65				stress resp.
SRV_02965_at	NP_006519	TFPI2	2.22				stress resp.
SRV_12417_at	NP_006087	NDRG1	2.57				stress resp.
AH_at	NP_006588	HSPA8	1.52		1.67		stress resp.
SRV_01846_at	NP_003352	UMOD	2.27		2.00		stress resp.
SRV_01294_at	NP_002023	FTH1		1.67		2.01	stress resp.
SRV_01385_at	NP_002293	LECT2		3.93		7.38	stress resp.
SRV_01840_at	NP_003346	UCP2		1.53		2.13	stress resp.
SRV_11406_at	NP_002341	LYN		1.74		1.72	stress resp.
SRV_11583_at	NP_002960	MAPK12		2.04		1.92	stress resp.
SRV_07726_a_at	NP_001034485	MPEG1		1.77		2.27	stress resp.
SRV_04367_a_at	NP_064455	IGLL1				2.29	stress resp.
SRV_04710_at	NP_078884	NEIL1				1.93	stress resp.
SRV_10936_at	NP_000945	PTGDS				4.19	stress resp.
SRV_00002_s_at	NP_064455	IGLL1				2.09	stress resp.
SRV_00078_at	NP_064455	IGLL1				2.43	stress resp.
SRV_01199_a_at	NP_001822	CLU				2.36	stress resp.
SRV_02442_a_at	NP_005092	G1P2		2.56			stress resp.
SRV_02611_a_at	NP_005618	SGK		1.57			stress resp.
SRV_13637_a_at	NP_066362	IFITM3		1.51			stress resp.
SRV_01323_a_at	NP_002124	HMOX1	10.05	18.24	5.13	8.53	antioxidant
SRV_00444_a_at	NP_000572	GPX1	1.67	1.51			antioxidant
SRV_01423_a_at	NP_002412	MMP1	26.65	37.91	2.94	11.41	ECM remod.
SRV_01428_x_at	NP_002416	MMP10	22.86	30.96	2.87		ECM remod.
SRV_01430_at	NP_002418	MMP13	22.54	34.09	9.04	24.97	ECM remod.
SRV_01425_at	NP_002418	MMP13	19.64	23.46	3.10	2.60	ECM remod.
SRV_11420_at	NP_071405	MMP27	16.71	23.32		4.52	ECM remod.
SRV_02399_a_at	NP_004985	MMP9	15.22	23.05	3.51	15.69	ECM remod.
SRV_11423_at	NP_002418	MMP13	6.79	8.48		2.20	ECM remod.
SRV_11417_a_at	NP_002412	MMP1	6.42	8.48			ECM remod.
SRV_12016_at	NP_004521	MMP2			1.71	2.49	ECM remod.
SRV_02241_at	NP_004521	MMP2			1.66	2.34	ECM remod.
SRV_10485_at	NP_004521	MMP2				2.11	ECM remod.
SRV_00453_a_at	NP_000651	TGFB1	2.40	2.32		1.93	multifunctional
SRV_00328_at	NP_000387	CTSK	7.03	8.07	4.86	19.35	lysosome
SRV_00327_a_at	NP_000387	CTSK	3.72	4.73	2.94	8.23	lysosome
SRV_00326_a_at	NP_000387	CTSK	3.19	3.78	2.84	6.47	lysosome
SRV_02600_a_at	NP_001008530	LGMN	2.16	2.81	1.80	3.66	lysosome
SRV_05534_at	NP_796377	RAB7B	1.91	1.99		1.83	lysosome
SRV_02353_a_at	NP_004853	LITAF	1.88	2.29		1.75	lysosome
SRV_01134_at	NP_001684	ATP6V1B2	1.83	2.24		1.96	lysosome
SRV_02294_at	NP_004682	ATP6V0D1	1.73	2.33		2.73	lysosome
SRV_14287_a_at	NP_666023	CTSL	1.66	2.23		2.03	lysosome
SRV_02601_at	NP_001008530	LGMN	1.66	2.09	1.58	2.44	lysosome
SRV_05336_a_at	NP_001903	CTSL		1.75		1.69	lysosome
SRV_00294_s_at	NP_000299	PPGB		1.67		2.12	lysosome
SRV_01135_a_at	NP_001685	ATP6V0C		1.62			lysosome
SRV_01242_a_at	NP_001900	CTSD		1.62		2.25	lysosome
SRV_01559_a_at	NP_002769	PSAP		1.58		1.61	lysosome
SRV_00355_at	NP_000425	NEU1		1.57		1.87	lysosome
SRV_02141_at	NP_004306	ASAH1		1.56		2.15	lysosome
SRV_09661_at	NP_001805	CTSC		1.52		1.99	lysosome
SRV_01558_at	NP_001035931	PSAP				2.71	lysosome
SRV_00334_a_at	NP_001073279	GLB1				1.64	lysosome
SRV_10701_at	NP_000387	CTSK				1.58	lysosome

Fold-level change of upregulated genes that have functions associated with a wounding response (identified by microarray analysis).

### Early wound-healing response during limb regeneration

Previous studies have documented anatomical similarities between innervated and denervated limbs at early stages of regeneration [20,43,44]. This study shows that there are also many transcriptional similarities. This suggests that many aspects of the early wound-healing phase of limb regeneration are not dependent upon post-amputation, nerve-derived factors (Table 6). Instead, humoral immune and local tissue responses appear to be key. Many genes that are associated with wound-healing and tissue-repair activities including stress, inflammation, cell survival, immunity, and extracellular matrix remodeling were upregulated from baseline in 5 dpa limbs (Figure 2; Table 6). It is probably no coincidence that essentially all of the early stress-associated genes that were previously identified as significantly regulated (using the same *Ambystoma *GeneChip) during early spinal cord regeneration [37], and during the innate immune response of axolotls to a deadly viral pathogen [38], were also identified as significant in this study. Many of these genes appear to be expressed similarly in all vertebrates in response to stress, including *junb, irf1*, *hmox1*, *apoE*, *mmps*, *ptx3*, *gal3*, *gadd45g*, and *tgfb*. Additionally, significantly more 'extracellular' genes were identified at 5 dpa than expected by chance, including genes that code for matrix remodeling proteins and secreted molecules whose functions are associated with growth factor binding, cell signaling, survival, death, adhesion, migration, and proliferation. The early wound-healing response initiates local environmental changes of the injury site that are pivotal to subsequent phases of regeneration.

The 5 dpa time point was chosen in this study to identify critical nerve-dependent signaling events that are stimulated within the first few days of regeneration (see [11]). Comparison of DL5 and NR5 data revealed few overall gene expression differences. The 5 dpa time point captured many transcriptional responses that are induced by injury and/or amputation but relatively few that are associated with known or suspected neurotrophic signaling pathways. More comprehensive sampling and deeper sequencing is needed to detail early nerve-dependent transcriptional responses because several genes that are known to be nerve-responsive during the wound-healing phase were not identified in this study (for example *sp9*, [11]; *prrx1*, *tbx5*, [10]).

### Downregulation of genes associated with differentiated muscle

This study documented dramatic decreases in the relative abundance of mRNAs coding for skeletal muscle contractile proteins, including myosins, actins, actinins, titin, tropomyosins, and troponins. Many of these changes were also detected by 454 cDNA sequence analysis (Figure 4). This strong, muscle-specific transcriptional signature was observed because approximately half of uninjured forelimb nuclei in 7 to 9 cm axolotls, and likely more than half the cross-sectional area, derive from muscle [45]. It is unlikely that the downregulation of muscle genes is due to retraction of the muscle towards the shoulder because muscle transcripts are much more downregulated at 14 dpa than 5 dpa in both denervated and innervated limbs. Considering that limb tissue samples in this study included ~1 mm of undamaged tissue proximal to the amputation plane, the results suggest that injury to skeletal muscle induces tissue-wide loss of muscle contractile transcripts. It is possible that the decrease in muscle transcripts is due to muscle wasting, caused by a lack of mechanical stress. It is interesting to speculate that this response maybe associated with the degeneration or cellularization of multinucleated muscle fibers into mononucleated cells, which occurs in both denervated and innervated amputated limbs [41,46-50]. It is interesting to note that muscle-specific genes, including *actc1*, *actn2*, *atp2a2*, *my1pf*, *tncc*, *tnni2*, *tnni3*, are downregulated during early stages of mammalian skeletal muscle regeneration [51], and this is accomplished without blastema formation. Skeletal muscle regeneration in mammals and other vertebrates involves resident stem (satellite) cells [52,53]. It is possible that muscle-specific genes are downregulated as an integral step of a conserved, skeletal muscle regeneration program of vertebrates. Candidate transcriptional repressor genes were identified in this study including *id3 *[54], *tardbp *[55], *cnot *[56], *msx1 *[6,57] and *msx2*. It will be important in future studies to determine if the dramatic decrease in muscle transcripts is due to activation of muscle stem (satellite) cells, muscle loss, muscle dysfunction/wasting, or whether these transcriptional patterns have an active role in regeneration.

### Genes associated with epigenetic reprogramming and genomic stability

Blastema formation requires a large number of progenitor cells derived from quiescent stem cells or differentiated cell types. It is generally known that reprogramming of differentiated cells is accompanied by epigenetic changes such as histone and DNA modifications [58,59]. Few candidates have been identified previously as bringing about epigenetic changes necessary for cellular reprogramming during regeneration [60,61]. This study identified several genes whose functions are associated with epigenetic phenomena, including chromatin remodeling, DNA methylation, and transcriptional regulation. These include *uhrf1 *[62], *lmnb2*, *parp1 *[63], *thymopoietin *[64], and a gene with high sequence identity to SAM-dependent methyltransferases (Cluster_227434_Contig1; SRV_05867_a_at).

After cellular reprogramming and during limb outgrowth, blastemal cells undergo tremendous cell proliferation. During blastema cell proliferation, telomere lengths and overall genome stability must be maintained to prevent cell death. This study identified several candidate genes from NR14 limbs that are known to function in genome stability, telomere homeostasis, and DNA repair. These include *parp1 *[65], *hmgb2 *[66], *fen1 *[67], *aurka *[68], *aurkb *[69], and *pif1*, a DNA helicase that maintains genome stability and binds to telomerase from yeast to humans [70]. It is also important to note that many transcripts were identified from NR and DL limbs that code for retroelement components (Table 5; for example polyproteins, gag proteins, reverse transcriptases, and recombinases). Retrotransposons are normally transcriptionally silenced in differentiated somatic cells by epigenetic mechanisms, but become active upon changes in epigenetic status; these may also regulate nearby gene expression [71,72]. It is unclear if upregulation of retroelement transcripts affects genome stability and/or is necessary for regeneration.

### Genes associated with nerve-dependent blastema outgrowth

In this study, blastemas were not observed in NR5 limbs or DL14 limbs, and only formed on NR14 limbs. Nerve-dependent limb outgrowth occurs as a result of blastemal cell proliferation. Two early cell proliferation biomarkers, *umod *and *psca*, were identified as significantly different between NR5 and DL5 limbs. *umod *probably locates to the wound epithelium as it is upregulated in apical skin cells during thyroid hormone-induced metamorphosis of axolotl epidermis (unpublished data). It is possible that *psca *is a membrane receptor of blastema cells because it shows structural similarity to *prod1*, a surface protein that is implicated in proximal-distal positional identity of blastemal cells during newt limb regeneration [73]. Most of the cell proliferation biomarkers were identified at 14 dpa, when a blastema was present in NR limbs but absent in DL limbs. Thus, between 5 and 14 dpa, blastemal cells underwent considerable cell proliferation in the presence of nerves. Because the limb blastema continues to expand after 14 dpa, the blastema-specific genes that were identified in this study are probably transcribed at much higher levels at later time points. A clear signature of cell proliferation, including genes that function in the cell cycle, mitosis, and nucleotide synthesis, was observed in NR14 limbs. In contrast, these genes were slightly downregulated in denervated limbs at this time (Table 1; Figure 5). Thus, transcripts associated with cell proliferation are maintained at steady state (no increased proliferation) in NR and DL limbs for at least five days. This early, nerve-independent portion of the limb regeneration program may allow time for re-innervation of the injury site and production of nerve-derived molecules in sufficient quantity to initiate and sustain blastemal cell proliferation.

Multiple gene products have been hypothesized to be neurotrophic factors provided by nerves to sustain blastema cell proliferation. These include growth-promoting factors such as fibroblast growth factors [11,31], substance P [74], neuregulin [33], and transferrin [32]. Transferrin and neuregulin were sampled by 454 sequencing, but were not identified as differentially expressed by our analysis. *fgf8 *and *fgf10 *were screened out of the microarray analysis due to low expression, but post-hoc analysis suggested that both are upregulated in NR14 limbs compared to control and DL14 limbs (Additional file 7). Expression of these molecules is known to be nerve-dependent in blastemas of *Xenopus *[75] and axolotls [3,4,76]. Other molecules that have previously been associated with the blastema during limb regeneration were not included in our statistical analyses due to low hybridization intensity, but were later found to be differentially expressed in NR14 limbs, including *hoxd10*, *hoxa13*, *hoxa11*, and *msx1 *(Additional file 7). Recently, Kumar et al [34] identified a growth-promoting extracellular ligand (nAG) that rescues aspects of nerve dependency of limb regeneration in newts. Probesets for *nAG *are represented on the *Ambystoma *GeneChip and transcripts for this gene were sampled by 454 cDNA sequencing. *nAG *mRNA was transcribed at a high level in all tissues, but did not differ significantly between NR, DL, or control limbs. It is possible that the effects of *nAG *and other neurotrophic candidates are associated with quantitative variation of mRNA transcript abundances over fine temporal and spatial scales. Such variation would have been missed in this analysis of three time points and sampling of mRNAs from heterogeneous tissues. Furthermore, it is possible that the increase in nAG immunoreactivity observed by Kumar et al [34] is regulated at the level of translation and would not be observed using microarray or sequencing approaches. Overall, we found that several, but not all, genes previously shown to be directly downstream of neurotrophic factors are expressed at higher mRNA levels in the blastema versus control and denervated samples.

Singer [17] emphasized that the neurotrophic factor underlying nerve dependency was growth promoting and quantitative in effect. He showed that the trophic effect of nerves could be titred by surgically manipulating the number of nerves innervating the limb [16]. Other lines of research showed that nerve-derived factors were not necessary for traversing cell cycle checkpoints, as an equivalent number of presumptive blastema cells are initially observed to enter S phase in denervated and innervated limbs [21,77-79]. Instead, the neurotrophic factor appears to be necessary to complete the cell cycle and this may be non-trivial considering the cost of replicating a large salamander genome. These studies suggest that nerves either directly or indirectly provide limiting macromolecules that are needed to accomplish cell division. According to this reasoning and assuming a correlation between transcript and protein abundance for specific mRNA species, transcripts associated with nerve dependency would be expected to exceed baseline levels in NR limbs during regeneration, and decrease in abundance in DL limbs. This study identified many human-axolotl presumptive orthologs and anonymous axolotl transcripts that showed this pattern. For example, *psat *codes for a protein that regulates the second enzymatic step of the phosphorylated pathway in mammals, which produces L-serine [80]. PSAT expression (protein and mRNA) is high among cell types with high rates of proliferation, including cancer cell lines [81,82]. Strikingly, *psat *registered the largest mRNA abundance difference between NR14 and DL14 limbs among probesets on the *Ambystoma *GeneChip (8.2-fold change), and this result was verified by both 454 cDNA sequencing (NR14 = 20.1, DL14 = 3) and real-time PCR (8.37-fold change; data not shown). We also found that the third enzyme in the phosphorylated pathway, *psph*, was 3.58-fold higher in NR14 versus DL14 by real-time PCR (data not shown). PSAT and PSPH may have neurotrophic potential but more likely function to provide proliferating cells with a limiting and conditionally important substance (L-serine) that is required for synthesis of macromolecules, amino acids, and purines that are needed to accomplish mitosis [80]. The fact that macromolecular synthesis and cell proliferation are both depressed in denervated limbs [83-86] supports the idea that PSAT activity is associated with nerve-derived factors that contribute to blastema outgrowth.

### Comparison of microarray and cDNA sequencing approaches

Microarray analysis and 454 cDNA sequencing offer different advantages for dissecting complex biological processes. The strength of microarray analysis is the precision that this approach provides for estimating transcript abundances for a specific panel of genes. Such gene panels provide standardized markers for identifying shared and unique patterns of transcription among experiments. In turn, identification of such patterns provides systems-level insight. For example and as was discussed above, cross-experiment comparisons of axolotl transcription helped distinguish general stress responses from local regenerative responses. A relatively conservative fold level threshold (> 1.5-fold) was used in this study to identify significant genes in the microarray analysis and lowly expressed genes (in the lowest quartile) were removed from consideration. These conservative approaches potentially exclude important genes but likely discover 'real' transcriptional differences between samples. A lower threshold could be applied to extract more information from the dataset and future studies would likely benefit by increasing replication of GeneChips to detect significant differences for lowly expressed genes. With respect to 454 cDNA sequencing, two goals were accomplished: gene discovery and estimation of transcript abundance. However, accomplishment of both goals required a trade-off in the allocation of resources toward deep sequencing versus replication. Sequencing resources were used to deeply sequence Day 0, NR, and DL cDNA libraries instead of shallowly sequencing replicate libraries for these time points. This strategy identified thousands of new gene sequences that will greatly enrich future regeneration studies. Future studies will benefit from experimental designs that replicate deep sequencing of cDNA libraries and this will be possible as sequencing costs decrease. Still, it was encouraging to find in this study that 38% of genes that were identified as differentially expressed by 454 cDNA sequencing and found on the microarray GeneChip were identified as significant by both of these technologies, and transcript abundances for these genes were highly positively correlated.

## Conclusion

This study addressed the nerve dependency of limb regeneration by characterizing downstream cellular events that are affected when an intact nerve supply is removed from an amputated salamander limb. Microarray analysis showed that the early wound response is largely nerve-independent, but transcriptional profiles diverge between denervated and innervated limbs when the innervated limb starts to regenerate. Pyrosequencing supported these microarray results while substantially increasing sequence information from the salamander transcriptome. This study shows the utility of next-generation sequencing platforms for gaining transcriptome information [87-89]. This new DNA sequence information will greatly enrich future regeneration studies using the axolotl.

## Methods

### Animal procedures

Mexican axolotls were obtained from the *Ambystoma *Genetic Stock Center at the University of Kentucky. Siblings were reared individually under *ab libitum *conditions to 60 to 70 mm snout to vent length. The 3^rd^, 4^th^, and 5^th ^spinal nerves that enter the left limb were severed at the brachial plexus behind the shoulder. Denervated left and innervated right limbs were amputated at mid-stylopod and allowed to regenerate for 5 and 14 days. Animal care and use procedures were approved by the University of Kentucky Internal Animal Care and Use Committee.

### Histology

Limbs were collected at 5 and 14 dpa and fixed in 4% paraformaldehyde, 1× PBS overnight at 4°C. Tissues were cryoprotected in sucrose, embedded in TissueTek, and sectioned at 16 μm. Eosin Y and Gill's hematoxylin #2 (Sigma-Aldrich, St. Louis, MO, USA) were used to stain cytoplasm and nuclei. DIC and brightfield images were taken on an Olympus AX80 microscope (Center Valley, PA, USA).

### RNA extraction and microarray analysis

DL and NR limbs were collected approximately 1 mm proximal to the amputation plane 5 dpa and 14 dpa. Nine animals were used for each time point and limbs were pooled into three groups of three. Left and right limbs were paired within animals when possible in making the pools for the 5 and 14 dpa time points. The day 0 pools were created using only the right limbs of nine different individuals that were collected within minutes following limb amputation. RNA was extracted using Trizol Reagent (Invitrogen, Carlsbad, CA, USA) followed by RNeasy minicolumns (Qiagen, Valencia, CA, USA). RNA quality was assessed by spectrophotometry using a Nanodrop ND-1000 (Nanodrop, Wilmington, DE, USA) and run on a Bioanalyzer 2100 (Agilent, Santa Clara, CA, USA). The *Ambystoma *microarray platform was produced by the Voss lab and Affymetrix and has been described elsewhere [35,37]. Total RNA was used to produce cRNA probes for GeneChip hybridizations (Affymetrix, Santa Clara, CA, USA) at the University of Kentucky Microarray Core Facility according to standard Affymetrix protocols. Probe level quality control analyses were performed as described in [37]. Data processing and statistical analysis was performed using the Affy Bioconductor package for the R statistical environment [90]. Background correction, normalization, and probe set summarization were performed via the robust multi-array average (RMA) algorithm of [91]. Correlation matrices (Pearson's *r*) for replicate GeneChips at the probe-set level were produced to assess correlation between GeneChips (minimum *r *= 0.9787, maximum *r *= 0.9952). Probe-sets were removed if mean signal intensity was less than the mean of the lowest quartile across all 15 GeneChips (mean ± standard deviation = 7.748 ± 0.031). Some microarray technologies may provide unreliable hybridization estimates for lowly expressed genes [92]. For this reason, a stringent cut-off was applied that removed the bottom quartile of genes for significance testing. Probe-set filtering yielded 3656 probe-sets for significance testing.

### Microarray analysis

The limma package [93,94] available from Bioconductor was used to conduct three analyses to statistically identify differentially expressed genes. In the first analysis, linear models were fit to each gene. These models used coefficients to denote each of the five treatments by sampling time combinations. The following coefficients were contrasted: Day 0 versus NR 5 dpa (NR5), Day 0 versus DL 5 dpa (DL5), Day 0 versus NR 14 dpa (NR14), Day 0 versus DL 14 dpa (DL14), NR5 versus NR14, and DL5 versus DL14. The other two analyses were equivalent to paired *t*-tests and compared NR5 versus DL5, and NR14 versus DL14. Multiple testing was corrected using an FDR cut-off of 0.05 and then a fold-change filter (≥ 1.5-fold change) was implemented to derive final gene lists. All microarray data are available at [95]. The identity of differentially expressed salamander transcripts was inferred from presumptive human orthologs. Orthology was assumed for all salamander transcripts that exhibited significant sequence similarity to protein coding sequences from human RefSeq and nr databases (BLASTx, *e *< 1 × 10^-7^). *K*-means gene clustering was performed using the Genesis software package [96]. Presumptive human-salamander orthologs were further annotated using GO terms and tools provided by the Database for Annotation, Visualization, and Integrated Discovery [97]. Significantly over-represented GO terms (EASE score *p *< 0.01) were identified for specific treatment/sampling time combinations. Certain GO terms were excluded from the results if similar information was represented by a similar GO term. The null expectation for GO term representation was obtained by assigning GO terms to 3271 EST contigs from *Ambystoma *ESTdb [95].

### 454 cDNA Sequence Analysis

The same total RNA samples that were used in the microarray analysis were used to produce cDNA templates for 454 pyrosequencing. cDNA libraries were generated for Day 0, NR5, NR14, DL5, and DL14 RNA samples using the Super SMART cDNA Synthesis protocol (Clontech, Mountain View, CA). Single-stranded cDNA template was amplified using the Advantage 2 PCR Kit (Clontech) and size selected according to manufacturer's instructions. cDNAs were sequenced using the Genome Sequencer FLX System (Roche Applied Science, Indianapolis, IN). SeqClean was used for vector/poor quality trimming, bacterial contaminant screening, and identification of *A. mexicanum *mitochondrial DNA and rDNA sequences [98]. Retained sequences were pre-clustered using PaCE and then assembled using CAP3 with a 90% sequence similarity threshold [99,100]. Contigs (including singletons) were searched using BLAST algorithms against the *Ambystoma *ESTdb, human and nr RefSeq databases, and *Xenopus laevis *and *X. tropicalis *Unigene sets. Annotated queries that returned a significant BLAST hit were assigned the gene identifier of the best-matching subject sequence. All of these new 454 sequence reads have been submitted to the Short Read Archive (SRA) at the National Center for Biotechnology Information (NCBI), accession SRA004195.2. 454 Sequences were assembled with previous EST data and are available at Sal-Site [95].

The number of times each 454 DNA sequence read matched a unique EST contig from the *Ambystoma *ESTdb was recorded, and these count data were used to estimate mRNA abundances for presumptive axolotl genes. The following method was used to identify differentially expressed genes among 10,275 contigs that were sampled ≥ 5 times across all five cDNA libraries. First, 5000 random draws were taken from a multinomial distribution to derive expected count data for each gene (*k *= 5 cDNA libraries, *n *= the sum of counts across all libraries for a given gene, and *p*_1_, *p*_2_, *p*_3_, *p*_4_, and *p*_5 _= expected proportion of counts per library given unequal sampling among cDNA libraries). Then, χ^2 ^statistics were calculated, on a gene-by-gene basis, for each of these random draws. *P*-values for each gene were estimated by calculating the proportion of randomized χ^2 ^statistics that were ≥ to the χ^2 ^statistic associated with the observed data. Contigs with *P*-values ≤ 0.001 were considered differentially expressed. Upon examination of this dataset, it was noticed that 589 of the significant EST contigs were uniquely derived from different cDNA libraries and these tended to form small (< 250 bp) contigs that did not match previous sequences in the *Ambystoma *ESTdb. These sequences were considered cloning/sequencing artifacts and removed from the dataset. This yielded a final dataset of 1150 significant genes. GO analyses were performed as described above with the exception that human default GO term frequencies were used to establish null expectations (EASE score *p *< 0.02).

## Authors' contributions

JRM and LGE performed surgeries, tissue collections, and RNA isolations. SP gave bioinformatic support and assembly of 454 sequences. RBP performed statistical analysis of microarray and 454 data. JRM and JAW performed histology, immunohistochemistry, and real-time PCR. SRV performed 454 sequencing template preparations. SRV and JRM drafted the manuscript. CKB, WZ, GMP, IMV, TH, SVB, DMG TTH, SRV, and JRM participated in the design of the study and helped draft the manuscript. All authors read and approved the final manuscript.

## Supplementary Material

Additional file 1 **371 unique probe sets upregulated from baseline (D0)**. The data show all the significant genes identified as upregulated from baseline. The blank cells represent insignificant results at particular time points. Gene functions were hand-annotated using gene ontology terminology, NCBI Entrez Gene descriptions, and Pubmed searches. Click here for file

Additional file 2 **336 unique probe sets that were downregulated from baseline (D0)**. The data show all the significant genes identified as downregulated from baseline. The blank cells represent insignificant results at particular time points. Gene functions were hand-annotated using GO terminology, NCBI Entrez Gene descriptions, and Pubmed searches. Click here for file

Additional file 3 **33 unique probe sets that were significantly different between NR5 and DL5**. The data shows the genes that were identified as differentially expressed between NR5 and DL5 limbs. Blank cells represent insignificant results at particular time points. Gene functions were hand-annotated using GO terminology, NCBI Entrez Gene descriptions, and Pubmed searches. Click here for file

Additional file 4 **282 unique probe sets that were significantly different between NR14 and DL14**. The data shows the genes that were identified as differentially expressed between NR14 and DL14 limbs. Blank cells represent insignificant results at particular time points. Gene functions were hand-annotated using GO terminology, NCBI Entrez Gene descriptions, and Pubmed searches. Click here for file

Additional file 5 **1150 contigs that differed significantly in abundance between cDNA libraries according to 454 sequencing**. The data show the normalized counts for genes that were identified as differentially expressed between any of the five libraries. Genes with significant human hits were hand-annotated using GO terminology, NCBI Entrez Gene descriptions, and Pubmed searches. Click here for file

Additional file 6 **104 genes that were represented on both platforms and found to be differentially expressed according to both microarry and 454 sequencing**. The data show the normalized counts for genes identified using 454 sequencing and fold changes for genes identified using microarray analysis. Gene functions were hand-annotated using GO terminology, NCBI Entrez Gene descriptions, and Pubmed searches. Click here for file

Additional file 7 **Lowly-expressed develeopmental genes from D14 limbs**. Important developmental genes that yielded weak, but significant hybridization signals between NR14 and DL14 limbs. Click here for file

## References

[B1] Dinsmore CE (1991). A history of regeneration research: Milestones in the evolution of science.

[B2] Carlson MR, Komine Y, Bryant SV, Gardiner DM (2001). Expression of Hoxb13 and Hoxc10 in developing and regenerating Axolotl limbs and tails. Dev Biol.

[B3] Christensen RN, Weinstein M, Tassava RA (2001). Fibroblast growth factors in regenerating limbs of *Ambystoma*: cloning and semi-quantitative RT-PCR expression studies. J Exp Zool.

[B4] Christensen RN, Weinstein M, Tassava RA (2002). Expression of fibroblast growth factors 4, 8, and 10 in limbs, flanks, and blastemas of *Ambystoma*. Dev Dyn.

[B5] Endo T, Bryant SV, Gardiner DM (2004). A stepwise model system for limb regeneration. Dev Biol.

[B6] Kumar A, Velloso CP, Imokawa Y, Brockes JP (2004). The regenerative plasticity of isolated urodele myofibers and its dependence on MSX1. PLoS Biol.

[B7] Mercader N, Tanaka EM, Torres M (2005). Proximodistal identity during vertebrate limb regeneration is regulated by Meis homeodomain proteins. Development.

[B8] Schnapp E, Kragl M, Rubin L, Tanaka EM (2005). Hedgehog signaling controls dorsoventral patterning, blastema cell proliferation and cartilage induction during axolotl tail regeneration. Development.

[B9] Levesque M, Gatien S, Finnson K, Desmeules S, Villiard E, Pilote M, Philip A, Roy S (2007). Transforming growth factor: beta signaling is essential for limb regeneration in axolotls. PLoS ONE.

[B10] Satoh A, Gardiner DM, Bryant SV, Endo T (2007). Nerve-induced ectopic limb blastemas in the axolotl are equivalent to amputation-induced blastemas. Dev Biol.

[B11] Satoh A, Graham GM, Bryant SV, Gardiner DM (2008). Neurotrophic regulation of epidermal dedifferentiation during wound healing and limb regeneration in the axolotl (*Ambystoma mexicanum*). Dev Biol.

[B12] Theodosiou M, Monaghan JR, Spencer ML, Voss SR, Noonan DJ (2007). Isolation and characterization of axolotl NPDC-1 and its effects on retinoic acid receptor signaling. Comp Biochem Physiol B Biochem Mol Biol.

[B13] Villiard E, Brinkmann H, Moiseeva O, Mallette FA, Ferbeyre G, Roy S (2007). Urodele p53 tolerates amino acid changes found in p53 variants linked to human cancer. BMC Evol Biol.

[B14] Ghosh S, Roy S, Seguin C, Bryant SV, Gardiner DM (2008). Analysis of the expression and function of Wnt-5a and Wnt-5b in developing and regenerating axolotl (*Ambystoma mexicanum*) limbs. Dev Growth Differ.

[B15] Todd TJ (1823). On the process of reproduction of the members of the aquatic salamander. J Sci Lit Arts.

[B16] Singer M (1952). The influence of the nerve in regeneration of the amphibian extremity. Q Rev Biol.

[B17] Singer M (1978). On the nature of the neurotrophic phenomenon in urodele limb regeneration. Am Zool.

[B18] Bryant SV, Endo T, Gardiner DM (2002). Vertebrate limb regeneration and the origin of limb stem cells. Int J Dev Biol.

[B19] Mescher AL (1996). The cellular basis of limb regeneration in urodeles. Int J Dev Biol.

[B20] Bryant SV, Fyfe D, Singer M (1971). The effects of denervation on the ultrastructure of young limb regenerates in the newt, *Triturus*. Dev Biol.

[B21] Mescher AL, Tassava RA (1975). Denervation effects on DNA replication and mitosis during the initiation of limb regeneration in adult newts. Dev Biol.

[B22] Geraudie J, Singer M (1981). Scanning electron microscopy of the normal and denervated limb regenerate in the newt, *Notophthalmus*, including observations on embryonic amphibia limb-bud mesenchyme and blastemas of fish-fin regenerates. Am J Anat.

[B23] Olsen CL, Barger PM, Tassava RA (1984). Rescue of blocked cells by reinnervation in denervated forelimb stumps of larval *Ambystoma*. Dev Biol.

[B24] Olsen CL, Tassava RA (1984). Cell cycle and histological effects of reinnervation in denervated forelimb stumps of larval *Ambystoma*. J Exp Zool.

[B25] Barger PM, Tassava RA (1985). Kinetics of cell cycle entry in innervated and denervated forelimb stumps of larval *Ambystoma*. J Exp Zool.

[B26] Ferretti P, Brockes JP (1991). Cell origin and identity in limb regeneration and development. Glia.

[B27] Irvin BC, Tassava RA (1998). Effects of peripheral nerve implants on the regeneration of partially and fully innervated urodele forelimbs. Wound Repair Regen.

[B28] Tassava RA, Olsen-Winner CL (2003). Responses to amputation of denervated *Ambystoma *limbs containing aneurogenic limb grafts. J Exp Zoolog A Comp Exp Biol.

[B29] Brockes JP, Kintner CR (1986). Glial growth factor and nerve-dependent proliferation in the regeneration blastema of Urodele amphibians. Cell.

[B30] Smith MJ, Globus M, Vethamany-Globus S (1995). Nerve extracts and substance P activate the phosphatidylinositol signaling pathway and mitogenesis in newt forelimb regenerates. Dev Biol.

[B31] Mullen LM, Bryant SV, Torok MA, Blumberg B, Gardiner DM (1996). Nerve dependency of regeneration: the role of Distal-less and FGF signaling in amphibian limb regeneration. Development.

[B32] Mescher AL, Connell E, Hsu C, Patel C, Overton B (1997). Transferrin is necessary and sufficient for the neural effect on growth in amphibian limb regeneration blastemas. Dev Growth Differ.

[B33] Wang L, Marchionni MA, Tassava RA (2000). Cloning and neuronal expression of a type III newt neuregulin and rescue of denervated, nerve-dependent newt limb blastemas by rhGGF2. J Neurobiol.

[B34] Kumar A, Godwin JW, Gates PB, Garza-Garcia AA, Brockes JP (2007). Molecular basis for the nerve dependence of limb regeneration in an adult vertebrate. Science.

[B35] Page RB, Monaghan JR, Samuels AK, Smith JJ, Beachy CK, Voss SR (2007). Microarray analysis identifies keratin loci as sensitive biomarkers for thyroid hormone disruption in the salamander *Ambystoma mexicanum*. Comp Biochem Physiol C Toxicol Pharmacol.

[B36] Page RB, Voss SR, Samuels AK, Smith JJ, Putta S, Beachy CK (2008). Effect of thyroid hormone concentration on the transcriptional response underlying induced metamorphosis in the Mexican axolotl (*Ambystoma*). BMC Genomics.

[B37] Monaghan JR, Walker JA, Page RB, Putta S, Beachy CK, Voss SR (2007). Early gene expression during natural spinal cord regeneration in the salamander *Ambystoma mexicanum*. J Neurochem.

[B38] Stewart JD, Storfer A, Page RB, Beachy CK, Voss SR (2008). Transcriptional response of Mexican axolotls to *Ambystoma tigrinum *virus (ATV) infection. BMC Genomics.

[B39] Deck JD (1961). The histological effects of partial denervation and amputation in larval salamander forelimbs. J Exp Zool.

[B40] Carlone RI, Mescher AL, Sicard RE (1985). Trophic factors from nerves. Regulation of Vertebrate Limb Regeneration.

[B41] Tank PW, Carlson BM, Connelly TG (1976). A staging system for forelimb regeneration in the axolotl, *Ambystoma mexicanum*. J Morphol.

[B42] Putta S, Smith JJ, Walker JA, Rondet M, Weisrock DW, Monaghan J, Samuels AK, Kump K, King DC, Maness NJ, Habermann B, Tanake E, Bryant SV, Gardiner DM, Parichy DM, Voss SR (2004). From biomedicine to natural history research: EST resources for *ambystomatid *salamanders. BMC Genomics.

[B43] Schotte OE, Butler EG (1941). Morphological effects of denervation and amputation of limbs in Urodele larvae. J Exp Zool.

[B44] Thorton CS (1953). Histological modifications in denervated injured forelimbs of *amblystoma *larvae. J Exp Zool.

[B45] Tank PW, Holder N (1979). The distribution of cells in the upper forelimb of the axolotl, *Ambystoma mexicanum*. J Exp Zool.

[B46] Hay ED (1959). Electron microscopic observations of muscle dedifferentiation in regeneration of *Amblystoma *limbs. Dev Biol.

[B47] Petrosky NS, Tassava RA, Olsen CL (1980). Cellular events in denervated limb stumps of Ambystoma larvae during re-innervation and subsequent regeneration. Experientia.

[B48] Lo DC, Allen F, Brockes JP (1993). Reversal of muscle differentiation during urodele limb regeneration. Proc Natl Acad Sci USA.

[B49] Kumar A, Velloso CP, Imokawa Y, Brockes JP (2000). Plasticity of retrovirus-labelled myotubes in the newt limb regeneration blastema. Dev Biol.

[B50] Echeverri K, Clarke JD, Tanaka EM (2001). In vivo imaging indicates muscle fiber dedifferentiation is a major contributor to the regenerating tail blastema. Dev Biol.

[B51] Goetsch SC, Hawke TJ, Gallardo TD, Richardson JA, Garry DJ (2003). Transcriptional profiling and regulation of the extracellular matrix during muscle regeneration. Physiol Genomics.

[B52] Morrison JI, Loof S, He P, Simon A (2006). Salamander limb regeneration involves the activation of a multipotent skeletal muscle satellite cell population. J Cell Biol.

[B53] Cameron JA, Hilgers AR, Hinterberger TJ (1986). Evidence that reserve cells are a source of regenerated adult newt muscle in vitro. Nature.

[B54] Iwasaki K, Hayashi K, Fujioka T, Sobue K (2008). Rho/Rho-associated Kinase Signal Regulates Myogenic Differentiation via Myocardin-related Transcription Factor-A/Smad-dependent Transcription of the Id3 Gene. J Biol Chem.

[B55] Buratti E, Baralle FE (2008). Multiple roles of TDP-43 in gene expression, splicing regulation, and human disease. Front Biosci.

[B56] Winkler GS, Mulder KW, Bardwell VJ, Kalkhoven E, Timmers HT (2006). Human Ccr4-Not complex is a ligand-dependent repressor of nuclear receptor-mediated transcription. EMBO J.

[B57] Schnapp E, Tanaka EM (2005). Quantitative evaluation of morpholino-mediated protein knockdown of GFP, MSX1, and PAX7 during tail regeneration in *Ambystoma mexicanum*. Dev Dyn.

[B58] Costa S, Shaw P (2007). 'Open minded' cells: how cells can change fate. Trends Cell Biol.

[B59] Maherali N, Sridharan R, Xie W, Utikal J, Eminli S, Arnold K, Stadtfeld M, Yachechko R, Tchieu J, Jaenisch R, Plath K, Hochedlinger K (2007). Directly reprogrammed fibroblasts show global epigenetic remodeling and widespread tissue contribution. Cell Stem Cell.

[B60] Yakushiji N, Suzuki M, Satoh A, Sagai T, Shiroishi T, Kobayashi H, Sasaki H, Ide H, Tamura K (2007). Correlation between Shh expression and DNA methylation status of the limb-specific Shh enhancer region during limb regeneration in amphibians. Dev Biol.

[B61] Palacios D, Puri PL (2006). The epigenetic network regulating muscle development and regeneration. J Cell Physiol.

[B62] Sharif J, Muto M, Takebayashi S, Suetake I, Iwamatsu A, Endo TA, Shinga J, Mizutani-Koseki Y, Toyoda T, Okamura K, Tajima S, Mitsuya K, Okano M, Koseki H (2007). The SRA protein Np95 mediates epigenetic inheritance by recruiting Dnmt1 to methylated DNA. Nature.

[B63] Guastafierro T, Cecchinelli B, Zampieri M, Reale A, Riggio G, Sthandier O, Zupi G, Calabrese L, Caiafa P (2008). CTCF activates PARP-1 affecting DNA methylation machinery. J Biol Chem.

[B64] Dorner D, Gotzmann J, Foisner R (2007). Nucleoplasmic lamins and their interaction partners, LAP2alpha, Rb, and BAF, in transcriptional regulation. FEBS J.

[B65] Shrivastav M, De Haro LP, Nickoloff JA (2008). Regulation of DNA double-strand break repair pathway choice. Cell Res.

[B66] Thomas JO (2001). HMG1 and 2: architectural DNA-binding proteins. Biochem Soc Trans.

[B67] Saharia A, Guittat L, Crocker S, Lim A, Steffen M, Kulkarni S, Stewart SA (2008). Flap endonuclease 1 contributes to telomere stability. Curr Biol.

[B68] Yang H, Ou CC, Feldman RI, Nicosia SV, Kruk PA, Cheng JQ (2004). Aurora-A kinase regulates telomerase activity through c-Myc in human ovarian and breast epithelial cells. Cancer Res.

[B69] Monaco L, Kolthur-Seetharam U, Loury R, Murcia JM, de Murcia G, Sassone-Corsi P (2005). Inhibition of Aurora-B kinase activity by poly(ADP-ribosyl)ation in response to DNA damage. Proc Natl Acad Sci USA.

[B70] Mateyak MK, Zakian VA (2006). Human PIF helicase is cell cycle regulated and associates with telomerase. Cell Cycle.

[B71] Kano H, Kurahashi H, Toda T (2007). Genetically regulated epigenetic transcriptional activation of retrotransposon insertion confers mouse dactylaplasia phenotype. Proc Natl Acad Sci USA.

[B72] Cropley JE, Martin DI (2007). Controlling elements are wild cards in the epigenomic deck. Proc Natl Acad Sci USA.

[B73] da Silva SM, Gates PB, Brockes JP (2002). The newt ortholog of CD59 is implicated in proximodistal identity during amphibian limb regeneration. Dev Cell.

[B74] Globus M, Smith MJ, Vethamany-Globus S (1991). Evidence supporting a mitogenic role for substance P in amphibian limb regeneration. Involvement of the inositol phospholipid signaling pathway. Ann N Y Acad Sci.

[B75] Suzuki M, Satoh A, Ide H, Tamura K (2005). Nerve-dependent and -independent events in blastema formation during *Xenopus *froglet limb regeneration. Dev Biol.

[B76] Han MJ, An JY, Kim WS (2001). Expression patterns of Fgf-8 during development and limb regeneration of the axolotl. Dev Dyn.

[B77] Tassava RA, Bennett LL, Zitnik GD (1974). DNA synthesis without mitosis in amputated denervated forelimbs of larval axolotls. J Exp Zool.

[B78] Maden M (1978). Neurotrophic control of the cell cycle during amphibian limb regeneration. J Embryol Exp Morphol.

[B79] Loyd RM, Connelly TG (1981). Microdensitometric analysis of denervation effects on newt limb blastema cells. Experientia.

[B80] de Koning TJ, Snell K, Duran M, Berger R, Poll-The BT, Surtees R (2003). L-serine in disease and development. Biochem J.

[B81] Martens JW, Nimmrich I, Koenig T, Look MP, Harbeck N, Model F, Kluth A, Bolt-de Vries J, Sieuwerts AM, Portengen H, Meijer-Van Gelder ME, Piepenbrock C, Olek A, Höfler H, Kiechle M, Klijn JG, Schmitt M, Maier S, Foekens JA (2005). Association of DNA methylation of phosphoserine aminotransferase with response to endocrine therapy in patients with recurrent breast cancer. Cancer Res.

[B82] Vié N, Copois V, Bascoul-Mollevi C, Denis V, Bec N, Robert B, Fraslon C, Conseiller E, Molina F, Larroque C, Martineau P, Del Rio M, Gongora (2008). Overexpression of phosphoserine aminotransferase PSAT1 stimulates cell growth and increases chemoresistance of colon cancer cells. Mol Cancer.

[B83] Dresden MH (1969). Denervation effects on newt limb regeneration: DNA, RNA, and protein synthesis. Dev Biol.

[B84] Lebowitz P, Singer M (1970). Neurotrophic control of protein synthesis in the regenerating limb of the newt, *Triturus*. Nature.

[B85] Singer M, Caston JD (1972). Neurotrophic dependence of macromolecular synthesis in the early limb regenerate of the newt, *Triturus*. J Embryol Exp Morphol.

[B86] Geraudie J, Singer M (1978). Nerve-dependent macromolecular synthesis in the epidermis and blastema of the adult newt regenerate. J Exp Zool.

[B87] Emrich SJ, Barbazuk WB, Li L, Schnable PS (2007). Gene discovery and annotation using LCM-454 transcriptome sequencing. Genome Res.

[B88] Toth AL, Varala K, Newman TC, Miguez FE, Hutchison SK, Willoughby DA, Simons JF, Egholm M, Hunt JH, Hudson ME, Robinson GE (2007). Wasp gene expression supports an evolutionary link between maternal behavior and eusociality. Science.

[B89] Vera JC, Wheat CW, Fescemyer HW, Frilander MJ, Crawford DL, Hanski I, Marden JH (2008). Rapid transcriptome characterization for a nonmodel organism using 454 pyrosequencing. Mol Ecol.

[B90] Bolstad B, Collin F, Brettshneider J, Simpson K, Cope L, Irizarry RA, Speed TP (2005). Bioinformatics and Computational Biology Solutions Using R and Bioconductor.

[B91] Irizarry RA, Hobbs B, Collin F, Beazer-Barclay YD, Antonellis KJ, Scherf U, Speed TP (2003). Exploration, normalization, and summaries of high density oligonucleotide array probe level data. Biostatistics.

[B92] Draghici S, Khatri P, Eklund AC, Szallasi Z (2006). Reliability and reproducibility issues in DNA microarray measurements. Trends Genet.

[B93] Smyth GK (2004). Linear models and empirical bayes methods for assessing differential expression in microarray experiments. Stat Appl Genet Mol Biol.

[B94] Smyth GK, Michaud J, Scott HS (2005). Use of within-array replicate spots for assessing differential expression in microarray experiments. Bioinformatics.

[B95] Sal-Site, an integrated web portal for the Ambystoma research community. http://www.ambystoma.org.

[B96] Sturn A, Quackenbush J, Trajanoski Z (2002). Genesis: cluster analysis of microarray data. Bioinformatics.

[B97] Dennis G, Sherman BT, Hosack DA, Yang J, Gao W, Lane HC, Lempicki RA (2003). DAVID: Database for Annotation, Visualization, and Integrated Discovery. Genome Biol.

[B98] The Computational Biology and Functional Genomics Laboratory at the Dana Farber Institute. http://compbio.dfci.harvard.edu/tgi/software/.

[B99] Kalyanaraman A, Aluru S, Kothari S, Brendel V (2003). Efficient clustering of large EST data sets on parallel computers. Nucleic Acids Res.

[B100] Huang X, Madan A (1999). CAP3: A DNA sequence assembly program. Genome Res.

[B101] Dahlquist KD, Salomonis N, Vranizan K, Lawlor SC, Conklin BR (2002). GenMAPP, a new tool for viewing and analyzing microarray data on biological pathways. Nat Genet.

